# Enhanced Oral Bioavailability of MT-102, a New Anti-inflammatory Agent, via a Ternary Solid Dispersion Formulation

**DOI:** 10.3390/pharmaceutics14071510

**Published:** 2022-07-21

**Authors:** Rajiv Bajracharya, Jae Geun Song, Sang Hoon Lee, Seong Hoon Jeong, Hyo-Kyung Han

**Affiliations:** College of Pharmacy, Dongguk University-Seoul, Dongguk-ro-32, Ilsan-Donggu, Goyang 10326, Korea; rajivbajra@hotmail.com (R.B.); songjguen@dongguk.edu (J.G.S.); sh_lee@dongguk.edu (S.H.L.); shjeong@dongguk.edu (S.H.J.)

**Keywords:** MT-102, solid dispersion, dissolution, indirubin, poloxamer 407, povidone K30

## Abstract

This study aimed to develop a solid dispersion (SD) of MT-102, a new anti-inflammatory agent, to improve its oral bioavailability. The ternary SD formulations of MT-102 (a poorly soluble extract of *Isatis indigotica* and *Juglans mandshurica*) were prepared using a solvent evaporation method with various drug/excipient ratios. Following that, the effectiveness of various SDs as an oral formulation of MT-102 was investigated using indirubin as a marker component. By forming SDs with hydrophilic polymers, the aqueous solubility of indirubin was significantly increased. SD-F4, containing drug, poloxamer 407 (P407), and povidone K30 (PVP K30) at a 1:2:2 weight ratio, exhibited the optimal dissolution profiles in the acidic to neutral pH range. Compared to pure MT-102 and a physical mixture, SD-F4 increased indirubin’s dissolution from MT-102 by approximately 9.86-fold and 2.21-fold, respectively. Additionally, SD-F4 caused the sticky extract to solidify, resulting in improved flowability and handling. As a result, compared to pure MT-102, the oral administration of SD-F4 significantly improved the systemic exposure of MT-102 in rats. Overall, the ternary SD formulation of MT-102 with a blended mixture of P407 and PVP K30 appeared to be effective at improving the dissolution and oral absorption of MT-102.

## 1. Introduction

Inflammatory bowel diseases (IBD), such as Crohn’s disease and ulcerative colitis, are chronic recurrent intestinal disorders characterized by transmural granulomatous inflammation or mucosa and submucosa inflammation [1]. Although the exact cause of IBD is unknown, genetic factors, host intestinal flora, and the host immune system may play a role in IBD pathogenesis [2]. Aminosalicylates, corticosteroids, anti-tumor necrosis factor-α antibodies, and immunosuppressants are currently being used to treat IBD [3]. However, the long-term use of these drugs has been linked to low efficacy and unfavorable side effects [4]. As a result, there is still an unmet need for more effective IBD drug therapy.

MT-102 is an herbal product extracted from *Isatis indigotica* and *Juglans mandshurica* that have been used to treat various diseases such as hepatitis, encephalitis, gastric ulcers, and diarrhea [5,6]. While MT-102 has shown efficacy in a mouse IBD model, it has low aqueous solubility, limiting its dissolution and oral bioavailability. Therefore, the solubilization of MT-102 is critical to improving its therapeutic potential and clinical application. Prodrugs, salt formation, micronization, solid dispersion, and lipid-based formulations have all been developed to improve the oral bioavailability of poorly soluble drugs [7,8,9]. Among them, solid dispersion (SD) is an effective method for increasing the solubility and dissolution of hydrophobic herbal products [10]. Previous studies have demonstrated that SDs significantly improved the dissolution and oral bioavailability of poorly soluble phytochemicals, such as curcumin and biochanin A [11,12]. Chen et al. [13] also demonstrated that amorphous SDs could improve the oral bioavailability of the main bioflavonoids from *Selaginella doederleinii* extract. Furthermore, Zhao et al. [14] compared the effect of SDs and self-emulsifying formulations on the dissolution and oral absorption of herbal extract from *Hippophae rhamnoides* L. The results indicated that SDs remarkably increased the oral bioavailability of three active components (isorhamnetin, quercetin, and kaempferol) in herbal extracts, but there was no significant increase in oral absorption via a self-emulsifying formulation in rats [14]. Overall, considering a simple preparation process and scale-up, cost-effectiveness, and drug loading capacity, an SD formulation should be an effective approach to enhancing the oral bioavailability of herbal extracts containing multiple insoluble components.

SD formulation comprises at least two different components, usually a hydrophilic polymer matrix and a hydrophobic drug, and the drugs are homogeneously dispersed in an amorphous or crystalline form into a polymer matrix, such as polyvinylpyrrolidone, polyethylene glycol, hydroxypropyl methylcellulose, or poloxamers [15]. Since various polymers available for SDs exhibit different physicochemical properties, functions, and safety profiles, the selection of appropriate polymeric carriers plays a vital role in the development of effective SD formulations. In general, polymeric carriers should be inert and compatible with active pharmaceutical ingredients. In addition, the physicochemical properties of polymers should be suitable for the preparation methods of SDs. From the kinetic perspective, polymeric carriers should be able to prevent or retard recrystallization of drugs in supersaturation conditions [15]. Furthermore, polymeric carriers with a high glass transition temperature (T*g*) may help improve the stability of SDs at room temperature [15,16,17,18,19]. In the kinetic and thermodynamic aspects, amphiphilic polymers (e.g., poloxamer 407 and Soluplus^®^) are advantageous for improving both the solubility and stability of amorphous SDs. They act as a surface-active agent, decreasing interfacial tension, increasing wettability, and effectively solubilizing the hydrophobic drugs via micelle formation [15,20,21]. At the same time, their polymeric nature helps stabilize a drug in an amorphous state [15].

**Figure 1:**
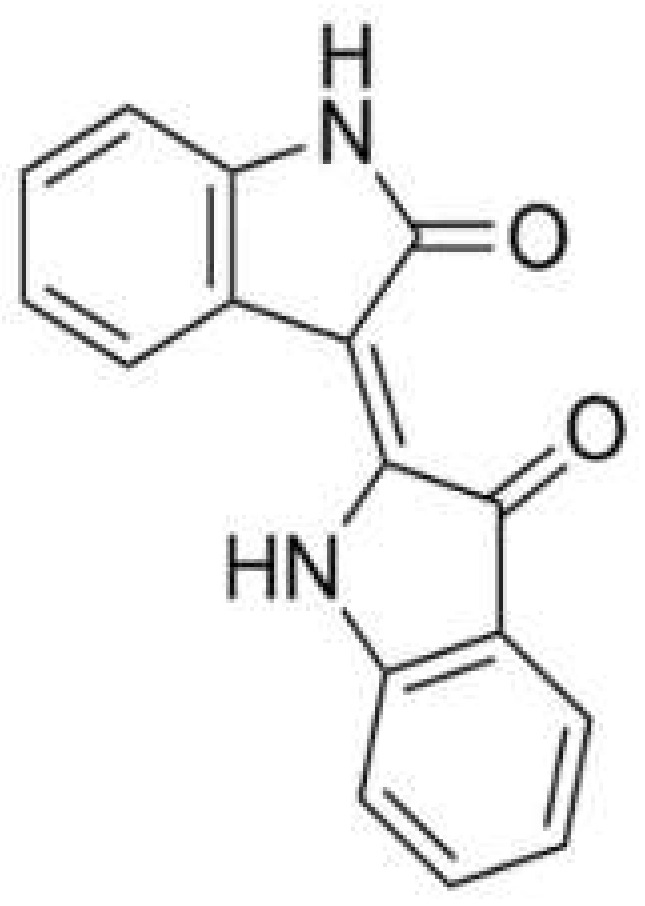
Structure of indirubin.

Given that a single polymer has a limited effect on preventing crystal growth and improving drug solubility, additional polymers or surfactants are often incorporated into binary drug–polymer systems to produce ternary or quaternary SDs [22]. For example, Prasad et al. [23] prepared the ternary SD of indomethacin with the combination of polymers (Eudragit E100 and PVP K90) and demonstrated that the ternary SD achieved higher stability and dissolution than the binary SD. Therefore, in this study, a ternary SD was developed as an effective oral formulation for improving the dissolution and bioavailability of MT-102. Different drug/excipient ratios were used to prepare ternary SDs of MT-102 using a solvent evaporation method. The effectiveness of various SDs as an oral formulation of MT-102 was then investigated using indirubin (Figure 1) as a marker component, since indirubin is a constituent of MT-102 and has anti-inflammatory properties [24]. The pharmacokinetic characteristics of the optimized SD formulation were also evaluated in rats. While Chen et al. [25] reported a self-micro-emulsifying drug delivery system (SMEDDS) that improved the oral absorption of indirubin, a marker of MT-102, they used indirubin as an isolated single compound. Therefore, this is the first report for an oral formulation improving the dissolution and oral absorption of MT-102, an herbal extract containing multiple components.

## 2. Materials and Methods

### 2.1. Materials

MT-102 was provided by MTHERA PHARMA (Seoul, Korea). Indirubin, 6-methoxy flavone, and (2-hydroxypropyl)-β-cyclodextrin were obtained from Sigma Aldrich (St. Louis, MO, USA). Poloxamer 188 (Kolliphor^®^, P188), poloxamer 407 (Kolliphor^®^, P407), povidone K30 (Kollidon^®^30, PVP K30), and copovidone K28 (Kollidon^®^ VA 64, Co-PVP) were provided by BASF-Korea (Seoul, Korea). Hydroxypropyl methyl cellulose E5 (Methocel^®^, HPMC E5) was obtained from Colorcon Asia Pacific PTE LTD. (Korea Branch, Suwon, Korea). Polyethylene glycol 6000 (PEG 6000) was obtained from the Daejung Chemical & Metal Co., Ltd. (Shiheung, Korea). Low-substituted hydroxypropyl cellulose (L-HPC) was purchased from Shin-Etsu (Tokyo, Japan). All the other chemicals were of analytical grade, and all the solvents were of high-performance liquid chromatography (HPLC) grade.

### 2.2. Screening of Carriers and Preparation of SDs

For the selection of optimal carriers, the SDs were prepared with various hydrophilic polymers at a drug–carrier ratio of 1:5 using the solvent evaporation method. Briefly, MT-102 and each polymeric carrier were dissolved in 70% (*v*/*v*) ethanol. After vigorous mixing at 2500 rpm for 2 min (Vortex-Genie 2, Cole-Parmer, Vernon Hills, IL, USA), all the solvents were removed under vacuum at room temperature. The resulting product was milled before being sieved through a 40-mesh screen. The carrier that increased the indirubin solubility the most was chosen for preparing the ternary SD formulations of MT-102. After selecting the suitable polymers, ternary SDs were prepared with various weight ratios of each component.

### 2.3. Solubility Studies for Carrier Selection

Each formulation (equivalent to 100 mg of MT-102) was added into water (1 mL) and equilibrated for 48 h at 37 °C with 100 rpm stirring. Samples were collected and filtered through a syringe filter (0.45 µm). The concentration of indirubin (a standard marker) in each sample was determined by HPLC assay.

### 2.4. In Vitro Drug Release Studies

For the selection of optimal SDs, the drug release profile of each formulation was evaluated in water. Each formulation (equivalent to 50 mg of MT-102) was filled into an empty hard gelatin capsule and spun at 100 rpm at 37 ± 0.5 °C in the dissolution medium (150 mL). The samples were collected at predetermined intervals and filtered through 0.45 µm pore-sized cellulose filters. The concentration of indirubin in each filtrate was determined by HPLC assay. For the characterization of the selected optimal formulation, the dissolution studies of SD-F4 and pure extract were carried out in buffer solution at different pH levels (1.2, 4.5, and 6.8) following the procedures described above. 

### 2.5. Morphology

The morphological characteristics of the optimized SD (SD-F4) were examined using a field emission scanning electron microscope (FE-SEM). The samples were spread out on a specimen stub, coated with platinum, and examined using an SEM (SU-70, Hitachi, Tokyo, Japan) at an acceleration voltage of 20 kV.

### 2.6. Stability Studies 

The SD-F4 formulation was placed in airtight vials and stored at 4 °C or 25 °C to test storage stability. After being stored for 1, 2, or 3 months, the samples were collected and underwent dissolution studies in water for 8 h to examine whether the dissolution characteristics of the SD-F4 formulation had changed. During storage, the solubility and morphological characteristics of SD-F4 were also investigated.

### 2.7. Pharmacokinetic Studies in Rats

The pharmacokinetic profile of SD-F4 was examined in rats. The study protocol for animal studies was approved by the review committee of Dongguk University (IACUC-2022-006-01). Male Sprague–Dawley rats (250–290 g) were supplied by Orient bio Co., Ltd. (Seongnam, Korea). All rats were given free access to tap water and a standard chow diet (Superfeed Company, Wonju, Korea) and were kept at a constant temperature of 21–22 °C with a 12 h light/dark cycle. The rats were divided into two groups (*n* = 5 per group) and fasted for 12 h prior to the experiments. They were given orally either an aqueous dispersion of pure extract (MT-102) or SD-F4 (equivalent to 500 mg/kg of MT-102). Blood samples were obtained from the femoral artery at the predetermined time points. The blood samples were centrifuged at 13,000× *g* for 5 min, and the resulting plasma samples were frozen at −20 °C until HPLC analysis. 

### 2.8. Analytical Methods

In vitro samples: Indirubin concentrations were determined using an HPLC system (Ultimate 3000 HPLC; Thermofisher, Waltham, MA, USA), which included a UV detector, a pump, and an automatic injector. A reversed-phase C18 column (Gemini C18, 4.6 mm × 150 mm, 5 µm; Phenomenex, Torrance, CA, USA) was eluted with a mobile phase of methanol and water (70:30, *v*/*v*). At 30 °C, the flow rate was 1.0 mL/min, and the UV wavelength was 289 nm. The internal standard (IS) was 6-methoxyflavone, and the calibration curve was linear (*r*^2^ = 0.99) in the concentration range of 0.02–2 µg/mL.

In vivo samples: A plasma sample (100 μL) was mixed with 6-methoxyflavone (IS: 20 μL, 25 μg/mL) and vortexed for 3 min. Then, 180 μL of methanol was added and vigorously mixed, followed by centrifugation at 13,000× *g* for 5 min. The supernatant was dried under vacuum. The residue was reconstituted with 80 μL of mobile phase and subjected to HPLC analysis. Chromatographic separation was conducted using a gradient elution of a mobile phase through a C18 column (Gemini C18, 4.6 mm × 150 mm, 5 µm; Phenomenex, Torrance, CA, USA) at 30 °C and a flow rate of 1.0 mL/min. The mobile phases comprised methanol (A) and water (B). The following were the gradient elutions: 0–1 min: 5% A and 95% B; 1–7 min: 30% A and 70% B; 7–12 min: 70% A and 30% B; 12–25 min: 70% A and 30% B; 25–30 min: 100% A; and 30–35 min: 5% A and 95% B. The calibration curves were linear over a concentration range of 0.01–10 µg/mL (*r*^2^ = 0.99).

### 2.9. Pharmacokinetic and Statistical Analysis

The area under the plasma concentration–time curve (AUC) was calculated using the linear trapezoidal method based on the non-compartmental analysis. The maximum plasma concentration (*C_max_*) and the time to reach the maximum plasma concentration (*T_max_*) were directly observed values from the experimental data.

The data are presented as the mean values with the standard deviation. Statistical analysis was performed using one-way ANOVA, followed by Dunnett’s test. A *p*-value < 0.05 was considered statistically significant.

## 3. Results and Discussions

### 3.1. Selection of Excipients

**Figure 2:**
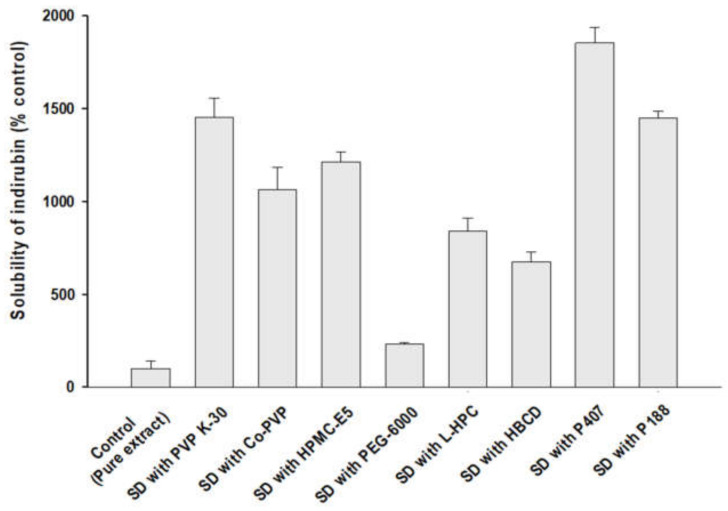
Effect of carriers on the aqueous solubility of indirubin in SD formulations of MT-102 (mean ± s.d., *n* = 3).

SD formulations of MT-102 were prepared with various polymeric carriers at a weight ratio of 1:5 using the solvent evaporation method to examine the effects of polymers on the solubilization of MT-102. Then, in each SD formulation, the aqueous solubility of indirubin (a standard marker of MT-102) was determined and compared to that of pure MT-102. As shown in Figure 2, all the polymer-based SDs enhanced the aqueous solubility of indirubin, although to different extents. Among the tested carriers, povidone K30 (PVP K30) and poloxamer 407 (P407) enhanced indirubin solubility the most, by about 15–18 fold compared to pure MT-102 (Figure 2). This result may be explained by better wettability, reduced particle size, and a change in drug crystallinity [8,26]. In general, adding a surfactant to an SD formulation should lower the degree of supersaturation, preventing nucleation and thermodynamic crystal growth, and thus inhibiting drug precipitation while improving drug dissolution [27]. Given that P407 has the dual function of an amphiphilic surfactant and a polymeric carrier [28], P407 can form micelles and allocate hydrophobic drugs into the micellar core [7,26,27], resulting in improved drug solubility. Next to P407, PVP K30 achieved the second highest enhancement in drug solubility (Figure 2), which may be due to an increase in wettability, dispersion of drugs in amorphous forms, and inhibition of recrystallization [27]. As a commonly used hydrophilic polymer, PVP K30 is soluble in volatile solvents and suitable for the solvent evaporation method. Furthermore, it has a high glass transition temperature (*T_g_* = 163 °C), which aids in the physical stability of amorphous SDs during storage [15,28]. Although P188 and PVP K30 showed solubility enhancement to a similar extent, PVP K30-based SDs provided a solid powder with better flowability, while P188-based SD was slightly viscous. Therefore, P407 and PVP K30 were selected to prepare the ternary SDs for MT-102 in this study. 

### 3.2. Optimization of SD Formulations

**Figure 3:**
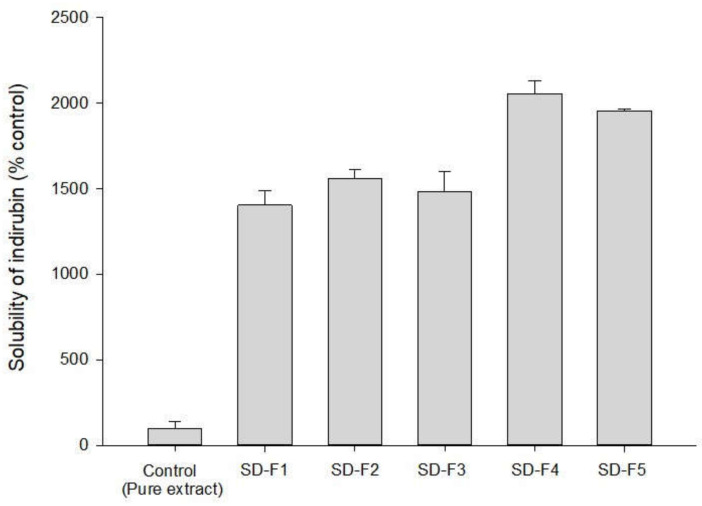
The effects of drug–polymer ratios on the aqueous solubility of indirubin in SD formulations of MT-102 (mean ± s.d., *n* = 3).

As summarized in Table 1, the ternary SD formulations (F1–F5) were prepared with P407 and PVP K30 at various drug–polymer ratios. The SDs effectively improved the solubility of indirubin compared to pure MT-102 (Figure 3). SD-F4-containing drug, P407, and PVP K30 at a 1:2:2 weight ratio increased the solubility of indirubin by 20-fold compared to pure MT-102.

**Figure 4:**
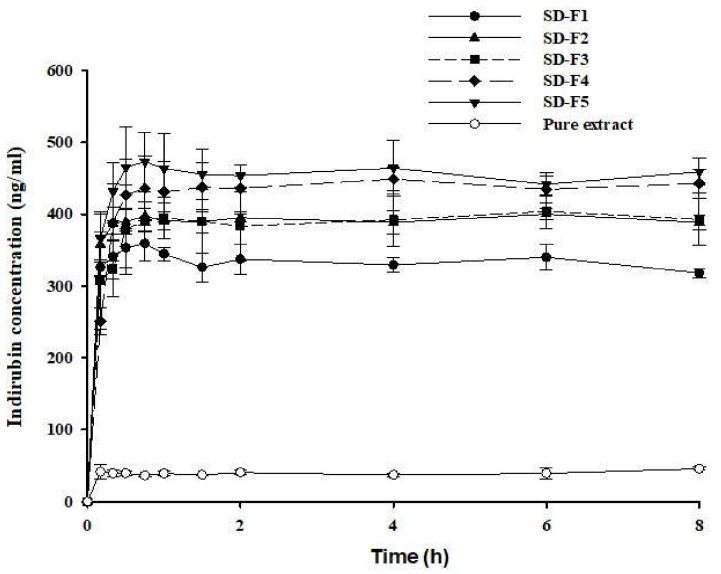
Dissolution profiles of indirubin from various SD formulations and pure extract (pure MT-102) in water (mean ± s.d., *n* = 3).

Additionally, the drug release profiles of the SD formulations were investigated in water. Figure 4 shows that the SDs significantly improved the dissolution of indirubin compared to pure extract. In particular, SD-F4 showed a rapid dissolution of indirubin within 1 h and dramatically increased the extent of drug dissolution. When compared to pure MT-102, SD-F4 increased the dissolution of indirubin by 9.86-fold. This could be due to various factors, including improved particle wettability, micellar solubilization of drugs, and the inhibition of recrystallization in the presence of hydrophilic carriers. However, when compared to SD-F4, further increasing the drug–polymer ratio to 1:3:2 (SD-F5) had no discernible effect on dissolution behavior. This phenomenon could be explained, at least in part, by the reversible gelling properties of P407. Increasing the P407 concentration can increase the viscosity via the complex physical entanglement, retarding drug diffusion and release from the polymeric matrix [29]. Taken together, the SD-F4 formulation was selected as the optimal SD formulation for MT-102.

**Figure 5:**
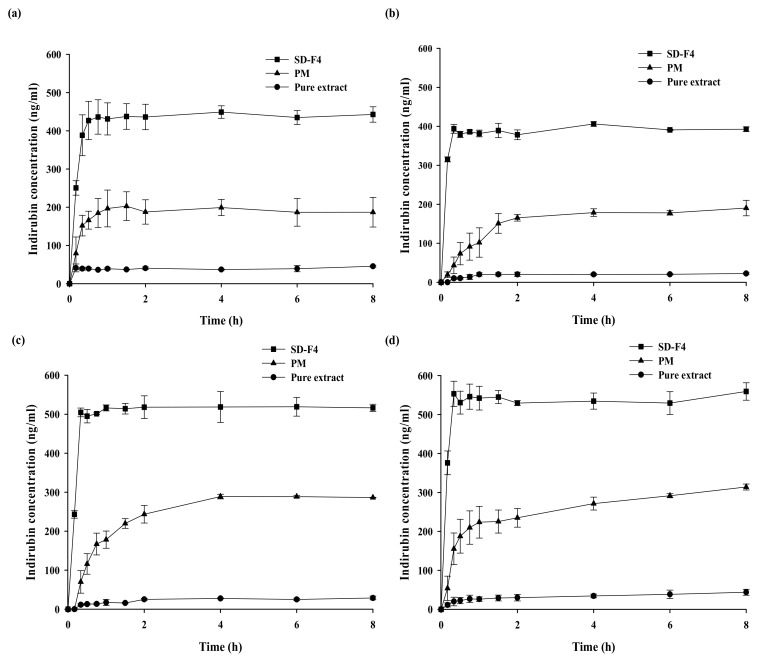
Dissolution profiles of indirubin from SD-F4, pure extract (pure MT-102), and physical mixture (PM) at various pH levels (mean ± s.d., *n* = 3). (**a**) Water, (**b**) pH 1.2, (**c**) pH 4.5, and (**d**) pH 6.8.

**Figure 6:**
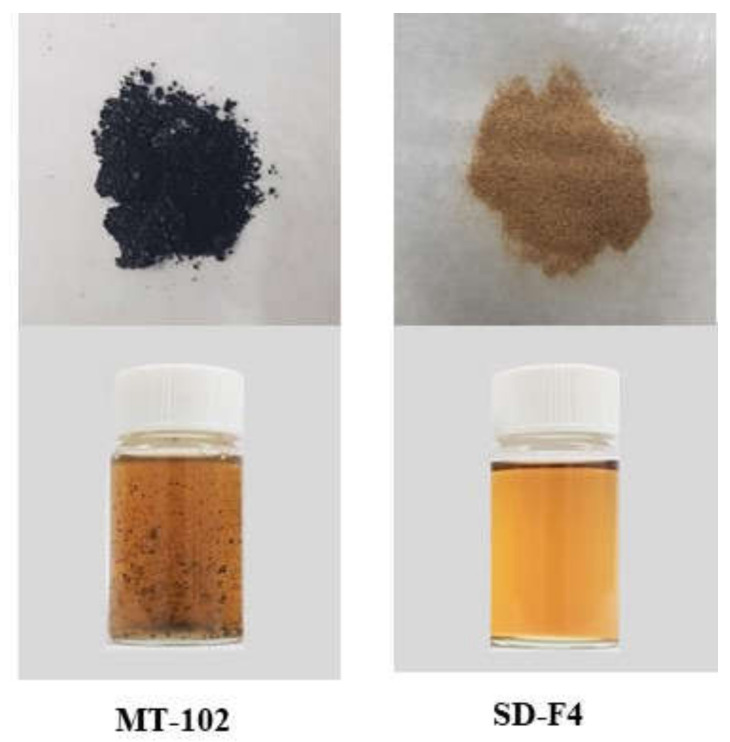
Images of MT-102 (pure extract) and SD-F4. Each formulation equivalent to 25 mg/mL of MT-102 was dissolved in water.

From the acidic to the neutral pH range, the pH dependency on drug dissolution from the optimal SD formulation (SD-F4) was investigated. As shown in Figure 5, SD-F4 significantly increased the rate and extent of drug dissolution, achieving a similarly high and fast drug release at all tested pH levels from acidic to neutral. This finding suggests that SD-F4 can maintain its dissolution properties as it travels through the gastrointestinal tract. Furthermore, when compared to its physical mixture, SD-F4 showed 2.21-fold higher drug dissolution, implying that drug crystallinity may be changed to an amorphous state in SD formulation. SD-F4 is a solid powder with better flowability and handling properties than the pure viscous extract (Figure 6). Additionally, it should be better suited to developing solid dosage forms, such as tablets and capsules, resulting in improved patient compliance. 

### 3.3. Storage Stability

**Figure 7:**
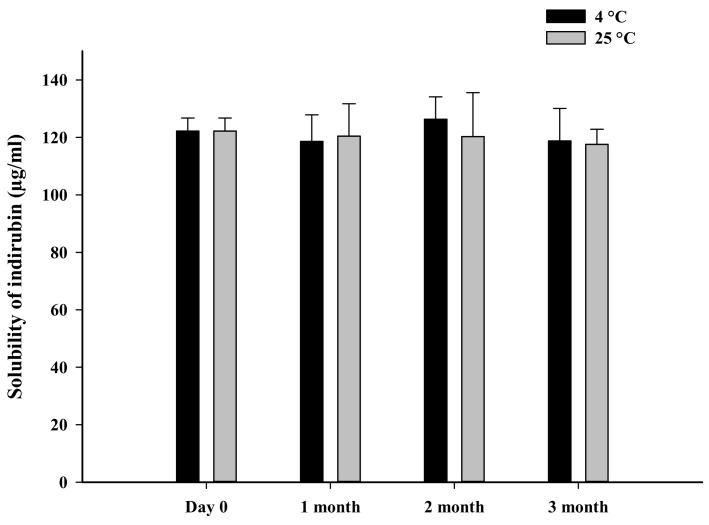
Solubility of indirubin in SD-F4 after three months of storage at 4 °C and 25 °C (mean ± s.d., *n* = 3).

**Figure 8:**
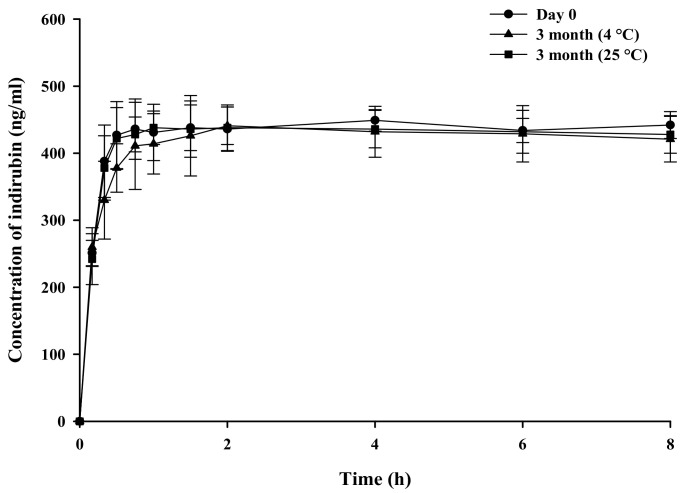
Dissolution profiles of indirubin from SD-F4 (mean ± s.d., *n* = 3). After three months of storage at 4 °C and 25 °C, the dissolution profiles of SD-F4 were evaluated in water and compared to those on Day 0.

The solubility and dissolution characteristics of SD-F4 were assessed after three months of storage at 4 °C and 25 °C. The enhanced solubility of indirubin was well maintained after storage at both temperatures, as shown in Figure 7. Additionally, the dissolution behavior of SD-F4 did not change during storage at 4 °C and 25 °C (Table 2 and Figure 8). This result indicates that drugs were stably dispersed in SD-F4, retaining the improved dissolution properties during storage. 

**Figure 9:**
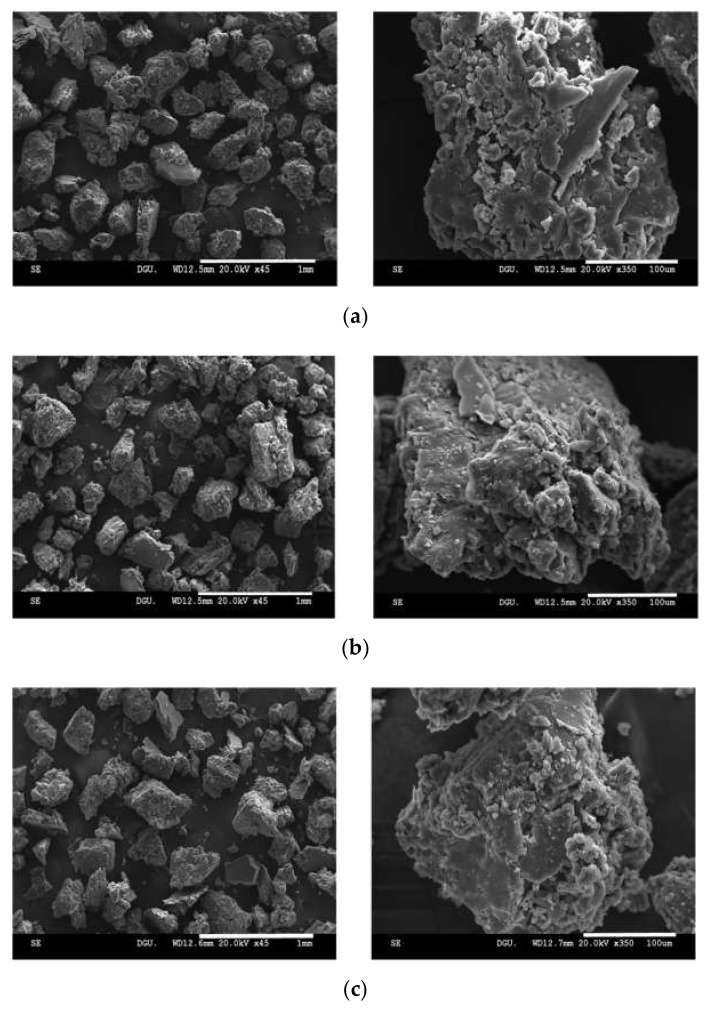
Morphology of SD-F4 on Day 0 (**a**) and after three months of storage at 4 °C (**b**) and 25 °C (**c**). The scale bar on the left panel is 1 mm, and the scale bar on the right panel is 100 µm.

The morphological characteristics of SD-F4 were also examined using scanning electron microscopy (SEM) after three months of storage. On Day 0, the SDs showed a homogeneous blend of all ternary components in irregular-shaped particles, as shown in Figure 9. The morphology of SD-F4 appeared to be well-maintained in the solid state after three months of storage, without aggregation.

### 3.4. Pharmacokinetic Study

**Figure 10:**
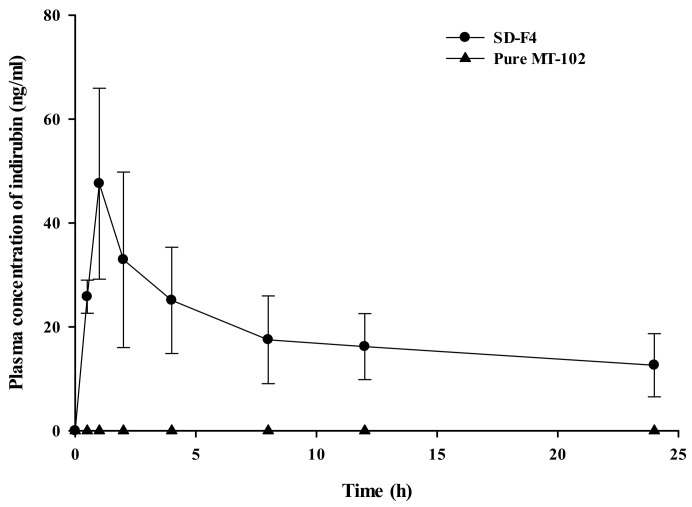
Plasma concentration–time profiles of indirubin after oral administration of SD-F4 or pure MT-102 to rats (mean ± s.d, *n* = 5). The dose was equivalent to 500 mg/kg of MT-102.

The oral pharmacokinetic profiles of SD-F4 and pure MT-102 were examined in rats. After the oral administration of MT-102 (pure extract), the plasma concentration of indirubin was so low that the pharmacokinetic parameters could not be determined. This result is consistent with the pharmacokinetic profile of indirubin reported by Chen et al. [25]. In contrast, the SD-F4 formulation improved the oral absorption of MT-102 and resulted in significantly higher systemic exposure of indirubin than pure MT-102 (Figure 10). SD-F4 also had a fast drug absorption, with a *T_max_* of less than 1 h (Table 3). These results could be due to the improved solubility and rapid dissolution of indirubin via the SD-F4 formulation. The amorphous drug dispersion in SD-F4 facilitated the rapid drug dissolution in the GI tract and increased luminal drug concentration, leading to the fast and enhanced drug absorption. In addition, P407, an amphiphilic polymeric carrier in SD-F4, increased the interface wetting and micellar solubilization of drugs, promoting intestinal drug absorption. Furthermore, the polymeric carriers in SD-F4 may increase the mucosal permeability of the intestinal epithelium, contributing to improved drug absorption [30,31,32]. Chen et al. [25] developed SMEDDS formulations using an isolated indirubin (single component) to improve oral bioavailability. In their study, following an oral administration of SMEDDS in rats, the *C_max_* and AUC of indirubin were 46.58–60.32 ng/mL and 499.64–681.69 µg·h/L, respectively. Considering that the indirubin content in MT-102 extract is approximately 0.16%, SD-F4 showed much higher oral exposure to indirubin than the SMEDDS in the literature when the pharmacokinetic data are normalized by the indirubin amount dosed to rats. However, due to the many variables in the experimental conditions, the direct comparison of pharmacokinetic data obtained from an isolated single compound and natural extract may not be appropriate. MT-102 is an herbal extract containing multiple active components, and SD-F4 can dissolve all of them, providing a clear aqueous solution of MT-102. Therefore, in addition to indirubin, SD-F4 may improve the oral absorption of other poorly soluble active components in MT-102, maximizing the synergistic effect in the therapeutic outcome. Taken together, SD-F4 was effective at improving the oral absorption of MT-102 in rats.

## 4. Conclusions

A ternary SD formulation of MT-102 (SD-F4) was prepared with PVP K30 and P407. In vitro and in vivo studies using indirubin as a marker component suggest that SD-F4 effectively improved the dissolution and oral absorption of poorly soluble MT-102. Given that herbal extract contains multiple active and insoluble components, the complete dissolution of MT-102 extract via SD-F4 may maximize the synergistic effect of multiple components in therapeutic efficacy. Furthermore, SD-F4 is a solid powder with better flowability and handling properties than pure viscous extract and may be more suitable for solid dosage forms, such as tablets and capsules, for improving patient compliance. Overall, the results suggest that SD formulation should be an effective approach to enhancing the oral bioavailability of herbal extracts containing multiple insoluble components.

## Figures and Tables

**Table 1:** Composition of ternary solid dispersions of MT-102.

Formulation	Composition (*w*/*w*/*w*)
SD-F1	Drug: P407: PVP K30 = 1:1:1
SD-F2	Drug: P407: PVP K30 = 1:1:2
SD-F3	Drug: P407: PVP K30 = 1:1:3
SD-F4	Drug: P407: PVP K30 = 1:2:2
SD-F5	Drug: P407: PVP K30 = 1:3:2

**Table 2:** Dissolution of indirubin from SD-F4 after storage at 4 °C and 25 °C (mean ± s.d., *n* = 3).

Temp. (°C)	Indirubin Concentration (ng/mL)			
	Day 0	1 Month	2 Months	3 Months
4	449 ± 16	436 ± 24	437 ± 22	441 ± 28
25	449 ± 16	438 ± 23	436 ± 26	438 ± 25

**Table 3:** Pharmacokinetic parameters of indirubin after oral administration of SD-F4 or pure MT-102 (pure extract) to rats (mean ± s.d., *n* = 5). The dose was equivalent to 500 mg/kg of MT-102.

Formulation	AUC (ng × h/mL)	*C_max_* (ng/mL)	*T_max_* (h)
SD-F4	448.5 ± 156.8	49.28 ± 15.43	0.9 ± 0.2
Pure MT-102	ND	ND	ND

ND: Not determined.

## Data Availability

Not applicable.
